# The biochemical dynamics of the glycogen phosphatase laforin directly impact brain metabolism

**DOI:** 10.1016/j.jbc.2025.111097

**Published:** 2025-12-22

**Authors:** M.Kathryn Brewer, Katherine J. Donohue, Pankaj K. Singh, Madushi Raththagala, Zoe R. Simmons, Jeremiah L. Wayne, Sheng Li, Rosa Viana, Dyann M. Segvich, Christopher J. Contreras, Alex R. Cantrell, Pascual Sanz, Ramon C. Sun, Craig W. Vander Kooi, Peter J. Roach, Anna DePaoli-Roach, Matthew S. Gentry

**Affiliations:** 1 Department of Molecular and Cellular Biochemistry, University of Kentucky College of Medicine, Lexington, Kentucky, USA; 2 Department of Biochemistry and Molecular Biology, University of Florida College of Medicine, Gainesville, Florida, USA; 3 Department of Chemistry, Skidmore College, Saratoga Springs, New York, USA; 4 Department of Medicine, University of California at San Diego, La Jolla, California, USA; 5 Instituto de Biomedicina de Valencia, CSIC and Centro de Investigación Biomedica en Red de Enfermedades Raras (CIBERER), Valencia, Spain; 6 Department of Biochemistry and Molecular Biology, Indiana University School of Medicine, Indianapolis, Indiana, USA

**Keywords:** Lafora disease, phosphatase, glycogen, glycogen storage disease, metabolomics

## Abstract

Laforin is the only known glycogen phosphatase. Mutations in the laforin gene lead to the fatal childhood dementia and progressive myoclonic epilepsy known as Lafora disease (LD). A hallmark of LD is aberrant, cytoplasmic, glycogen-like aggregates known as Lafora bodies. Surprisingly, recent reports indicate that overexpression of a phosphatase-deficient laforin mutant, with the catalytic cysteine mutated to serine (LCS), prevented the formation of Lafora bodies in a laforin KO mouse model. This finding led to questions regarding the biological relevance of laforin phosphatase activity and its role in LD etiology. In this study, we defined the *in vitro* and *in vivo* effects of the LCS mutation. LCS protein lacks catalytic activity but exhibits significantly higher binding to phosphate and long glucan chains compared with WT laforin. In addition, LCS exhibits altered dynamics *via* hydrogen–deuterium exchange mass spectrometry and interacts more robustly with its binding partners malin and protein targeting to glycogen. We demonstrate that these altered dynamics result in aberrant retention of the LCS protein in the brain of the LCS knock-in mouse model, compared with laforin levels in WT mice. To examine the metabolic consequences of these biophysical changes, we compared the brain metabolomic phenotypes of LCS mice to WT and laforin KO mice. Furthermore, LCS mice display a distinct and significant global perturbation in metabolism. These results indicate a key signaling role for glycogen phosphorylation in glycogen metabolism, revealing an important biological role for laforin catalytic phosphatase activity.

Mutations in the epilepsy, progressive myoclonic type 2A (*EPM2A*) gene, which encodes the glycogen phosphatase laforin, cause Lafora disease (LD) (1, 2). LD is a fatal childhood-onset epilepsy, dementia, and glycogen storage disorder (3, 4, 5, 6, 7). LD is characterized by the accumulation of aberrant glycogen that results in the formation of hyperphosphorylated, cytoplasmic, glycogen-like polyglucosan aggregates known as Lafora bodies (LBs) (8, 9, 10, 11). In the brain, LBs are abundant in astrocytes, observed in neurons, and are a hallmark of LD (10, 12, 13). In addition, we demonstrated that the brain metabolic profile in laforin KO (LKO) mice is distinctly different from WT mice (14, 15). Genetic knockdown or deletion of glycogen synthase or its activator protein targeting to glycogen (PTG) prevents LB formation, gliosis, and the neurological phenotypes in LD mouse models (16, 17, 18, 19, 20, 21). It was recently demonstrated that abnormal glycogen metabolism in astrocytes is a predominant driver of LD gliosis, whereas neuronal LBs contribute to LD epilepsy (15). Thus, both metabolic and cell type–specific factors contribute to LD, but it remains unclear if these are integrated or separable drivers of LD etiology.

Laforin is comprised of a dual-specificity phosphatase (DSP) domain and a carbohydrate-binding module from the CBM20 family (1, 2, 22). Laforin is the founding member of the glucan phosphatase family of enzymes, which all have a DSP domain and directly dephosphorylate complex carbohydrates (23, 24). DSPs belong to the protein tyrosine phosphatase (PTP) superfamily of enzymes that utilize a catalytic triad of cysteine, arginine, and aspartate residues (Cx_5_R) (25, 26). The PTP-loop contains the Cx_5_R-motif in which the cysteine functions as the catalytic nucleophile and the arginine stabilizes the phosphate moiety of the substrate (27). A flexible loop containing the catalytic aspartic acid, often called the D-loop, closes over the active site once the phosphosubstrate is bound, and acts as both a general acid and a base during catalysis. Glucan and lipid phosphatases are atypical DSPs, and their active site topologies are suited to their specific substrates (28, 29). The crystal structure of laforin (Protein Data Bank code: 4RKK) revealed that it forms an antiparallel dimer, with tight interdomain coupling (30). This organization allows an integrated allosteric coupling so that laforin can both bind the complex carbohydrate substrate and partner proteins (30, 31, 32, 33).

A catalytically dead laforin can be generated by mutating active site residues; the most common mutation involves mutating the catalytic cysteine to serine, that is, laforin C/S (C266S in human laforin and C265S in mouse laforin, both referred to as LCS hereafter) (34). The LCS mutant lacks phosphatase activity *in vitro* and therefore has been used as the primary negative control in studies of laforin function (23, 24, 30, 33, 35, 36, 37, 38, 39, 40, 41, 42). Surprisingly, it was reported that transgenic overexpression of LCS (either human or mouse) in LKO mice prevented LB formation (43, 44). Glycogen in the LKO mice expressing the mouse LCS transgene accumulated to WT levels, and branching was normal, but this glycogen was still hyperphosphorylated (44). These results suggest that laforin prevents LB formation, independent of its catalytic activity, raising questions about the necessity of the phosphatase function of laforin in LD development. However, in both previous reports, LCS, either human or mouse, was overexpressed up to 100-fold, and no analysis of neuropathology was performed. Recently, a knock-in mouse model of LCS was assessed, and it too did not exhibit LBs (45). However, it was concluded that the lack of LBs was multifactorial on a physiological level, and there were no biophysical analyses.

In the present study, we demonstrate that human LCS exhibits unique properties *in vitro* compared with WT laforin, behaving as a substrate trap. While the LCS mutation prevented catalysis, it significantly increased substrate and product binding. Using hydrogen–deuterium exchange (HDX) mass spectrometry (MS), we determined that the increased binding is due to altered conformational dynamics that also enhance interactions with its binding partners, malin and PTG, and displayed significantly increased protein levels in the brain. To probe the metabolic implications of these differences, we measured the effect of laforin mutation in the brain. Strikingly, LCS animals displayed a metabolic profile distinct from WT mice. These data indicate that laforin phosphatase activity contributes to maintaining brain metabolism, demonstrating a role for glycogen phosphorylation in metabolic signaling.

## Results

### Laforin mutant LCS binds phosphate and displays altered carbohydrate binding

**Figure 1:**
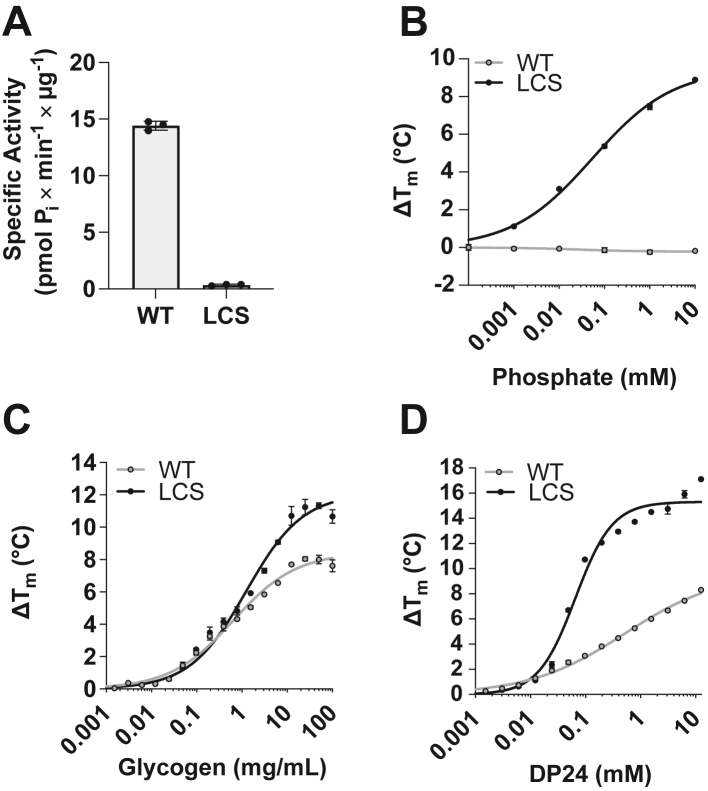
**LCS possesses unique binding properties to phosphate and glucans.***A,* specific activity of WT laforin and LCS using glycogen as a substrate, measuring released phosphate. *B,* phosphate binding curves determined by differential scanning fluorimetry (DSF). *C* and *D,* Δ*T*_m_ binding curves of WT *versus* LCS with glycogen (*C*) and DP24 (*D*) determined by DSF in the absence of added phosphate. All data represent the average ± SD of three technical replicates. LCS, laforin cysteine-to-serine mutant.

To examine differences between WT laforin and LCS, we first characterized biochemical differences between the proteins. Mutation of the active site cysteine to serine (C266S in human laforin) is one of the most common ways to produce a catalytically inactive PTP or DSP (34). As expected, the LCS mutant displayed complete loss of glycogen phosphatase activity (Fig. 1*A*). This result is consistent with the well-characterized PTP mechanism regarding the nucleophilic cysteine at the core of the catalytic triad (29, 30, 46, 47, 48, 49, 50).

We previously determined the structure of LCS in the presence of phosphate and maltohexaose (30). Although no phosphate was added to the purification or crystallization buffers, the structure revealed a phosphate molecule at each active site proximal to the C3 hydroxyl of the cocrystallized glucan, mimicking the product of the laforin dephosphorylation reaction. This phosphate scavenging suggested that LCS displays different binding properties compared with WT. To directly test this hypothesis, we assessed the ability of laforin to directly bind phosphate using differential scanning fluorimetry **(**DSF), a method used to define protein–ligand interactions. Apparent binding constants (*K*_*d*,app_) from these interactions can be calculated using DSF (51). Strikingly, LCS exhibited a concentration-dependent increase in Δ*T*_m_, shifting 8 °C with 10 mM phosphate, with a *K*_*d*,app_ of 55 ± 12 μM (Fig. 1*B*). In contrast, the *T*_m_ did not significantly shift for WT laforin, indicating WT laforin does not bind phosphate (Fig. 1*B*). Similarly, spectrophotometric experiments using malachite green also identified phosphate bound to recombinant LCS protein and not to WT laforin or the C266A laforin catalytic mutant (Fig. S1).

We next tested whether LCS displays an altered interaction with carbohydrate substrates. In the absence of substrate, WT laforin and LCS displayed similar *T*_m_ values of 49.2 ± 0.4 °C and 49.1 ± 0.2 °C, respectively (Fig. 1*C*). In the presence of glycogen, both WT laforin and LCS exhibited concentration-dependent binding and stabilization (Fig. 1*C*). LCS laforin exhibited a somewhat higher *K*_*d*,app_ compared with WT (3.0 mM *versus* 0.6 mM) and a much higher *B*_max,app_, that is, maximum Δ*T*_m_ (16.5 ± 0.78 °C *versus* 5.4 ± 0.22 °C) (Table S1). However, using an oligosaccharide mixture of approximately 24 α1,4-linked glucose residues (DP24), LCS displayed a much lower *K*_*d*,app_ (46 μM *versus* 410 μM) and a much higher *B*_max,app_ (Fig. 1*D*, Table S1). As we previously reported (30), the Hill coefficient for LCS binding to DP24 (h = 1.335) indicates a cooperative binding mechanism, and this was not observed with glycogen or WT laforin binding to either substrate (Table S1). These data indicate that compared with WT laforin, LCS exhibits significantly enhanced binding to both phosphate and long oligosaccharide substrates, both putative products of the laforin dephosphorylation reaction.

Although most glycogen sources contain low levels of phosphate, studies from LD models lacking laforin show that the aberrantly formed glycogen accumulating as LBs contains much higher phosphate and longer oligosaccharide chains, suggesting this long phosphorylated oligosaccharide chain is a laforin substrate (52). Therefore, we quantified how the presence of added phosphate affected the binding of WT laforin and LCS to glycogen and DP24. While phosphate did not affect WT binding to either substrate, 0.1 and 1 mM phosphate affected the interaction of LCS with both carbohydrates (Fig. S2, Table S1). Specifically, in the presence of DP24 with increasing phosphate, the *K*_*d*,app_ for LCS increased from 46 μM to 5.5 mM. Furthermore, the cooperative binding exhibited by LCS in the presence of DP24 was eliminated in 0.1 mM phosphate + DP24 (h = 0.99), and in 1 mM phosphate + DP24, negative cooperativity was observed (h = 0.56). The higher *K*_*d*,app_ and negative cooperativity with added phosphate suggest that phosphate binding to LCS reduces the affinity for subsequent DP24 binding.

### Conformational differences in LCS enhance protein–protein interaction

**Figure 2:**
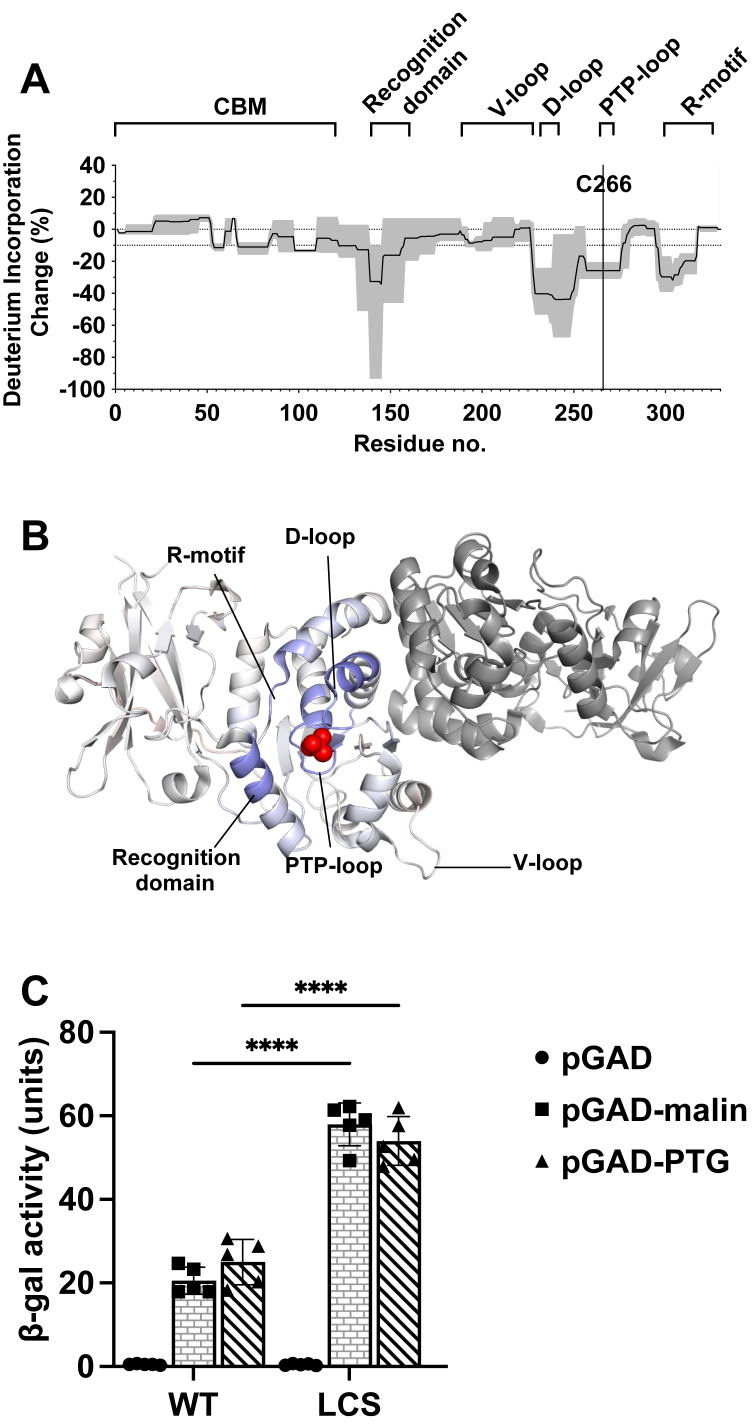
**LCS is less solvent accessible than WT laforin and interacts more strongly with binding partners.***A,* hydrogen–deuterium exchange mass spectrometry was performed on WT laforin and LCS. Change in deuterium incorporation in LCS compared with WT laforin was calculated based on overlapping peptides. Negative values indicate less deuteration in the LCS. *B,* deuteration changes were mapped onto the LCS crystal structure and color coded to show the extent of deuteration change. The active site is highlighted with the *red* phosphate molecule. DSP active site motifs are labeled in (*A*) and (*B*). *C,* binding of WT and LCS proteins to malin and PTG as determined by yeast two-hybrid assay. Each bar is average ± SD of 4 to 5 independent transformants and was analyzed by two-way ANOVA (row factor F [1, 24] = 219.7, *p* < 0.0001; column factor F [2, 24] = 302.3, *p* < 0.0001; interaction F [2, 24] = 57.88, *p* < 0.0001) followed by Tukey’s multiple comparisons test. ∗∗∗∗*p* < 0.0001. DSP, dual-specificity phosphatase; LCS, laforin cysteine-to-serine mutant.

HDX was previously utilized to define the dynamics of WT laforin and LD-causing missense mutations (30, 33). To compare the differential dynamics of WT laforin and LCS, we performed comparative HDX analysis. We identified a total of 310 high-quality matched peptides that covered 99.7% of residues (Fig. S3). LCS displayed significantly lower global conformational dynamics, as evidenced by decreased deuteration in many peptides compared with WT laforin. The most significant changes were observed in the classic motifs of the DSP family: the recognition domain (33% average reduction), the D-loop and its adjacent helix α8 (40–44% average reduction), the PTP-loop (26% average reduction), and the R-motif (Cx_5_R, 20–30% average reduction) (Fig. 2*A*). These differences in dynamics are highly spatially localized in the region surrounding the laforin active site (phosphate in *red*, Fig. 2*B*).

Studies of prototypical PTPs, such as PTP1B (a well-characterized phosphatase regulator of many cellular signaling processes) and YopH (a key virulence factor in pathogenic *Yersinia* species), show that the D-loop typically fluctuates between open and closed configurations, and catalysis is initiated by the closing of the D-loop *via* a ∼10 Å swinging motion (53, 54, 55, 56, 57). The C/S mutant of YopH was also previously analyzed by HDX and similarly displayed reduced deuteration in the D-loop, and molecular dynamics simulations suggested that in this mutant, the D-loop remains stably in the closed confirmation (54, 58). Our HDX results strongly suggest that the D-loop similarly favors a closed conformation in LCS, likely because of the presence of tightly bound phosphate in the active site. These data indicate that LCS likewise functions as a glycogen trap.

In addition to binding and dephosphorylating glycogen, laforin interacts with malin and other proteins associated with glycogen metabolism, including PTG (45, 59, 60, 61, 62, 63, 64). We previously showed that most LD-causing missense mutations negatively affect protein stability and/or binding to carbohydrates or other proteins (malin or PTG), suggesting these are all critical laforin functions that may be coupled (33). Surprisingly, we also demonstrated that many LD-causing mutations do not decrease the phosphatase activity of laforin. Since conformational differences in LCS detected by HDX were associated with changes to phosphate and carbohydrate binding, we next sought to measure the effect of LCS on interaction with malin and PTG. The malin and PTG interactions with laforin were previously defined *via* directed yeast two-hybrid assays (33, 59, 62, 65, 66, 67, 68, 69, 70, 71). LCS enhanced malin and PTG interactions by nearly twofold increased signal compared with WT laforin (Fig. 2*C*). These data demonstrate that the altered protein dynamics of LCS are associated with an increased interaction with phosphate, long oligosaccharides, malin, and PTG.

### Physiological consequence of differential LCS activity

**Figure 3:**
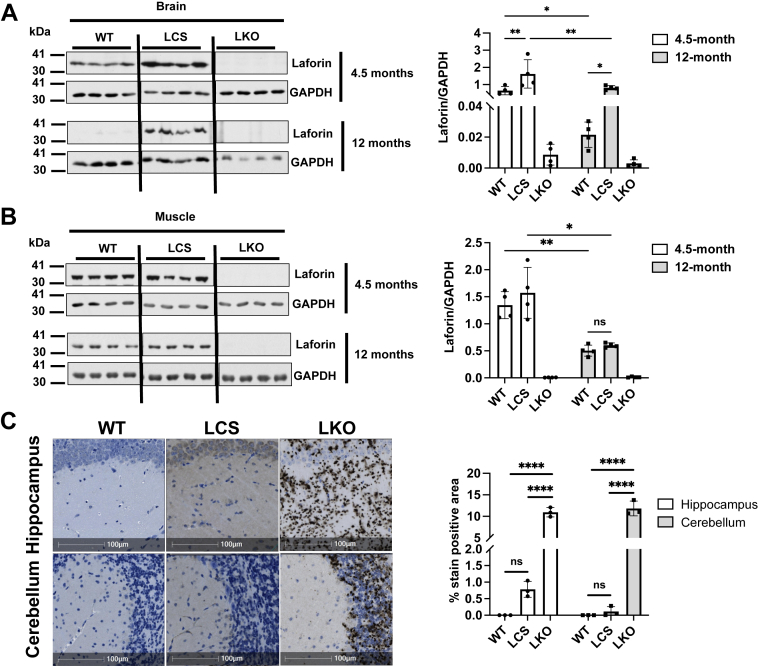
**LCS acquires differential *in vivo* stability and supports modest glycogen accumulation in the brain.** WT and LCS protein levels in the brain (*A*) and skeletal muscle (*B*) of 4.5-month and 12-month-old mice were determined by Western blot (n = 4 biological replicates/genotype). GAPDH was used as a loading control. Data are presented as mean ± SD, and *p* values were determined by two-way ANOVA (brain [A]—F [2, 9] = 23.5, *p* = 0.0003; interaction F [2, 9] = 2.919, *p* = 0.1054; skeletal muscle [B]—F [1, 6] = 54.68, *p* = 0.0003; interaction F [1.284, 7.7] = 10.34, *p* = 0.0102), followed by Tukey’s multiple comparisons test. ∗*p* < 0.05, ∗∗*p* < 0.005. *C,* anti-glycogen antibody (IV58B) staining of hippocampus and cerebellum from 12-month-old WT, LCS, and LKO mice to indicate glycogen load (the scale bar represents 100 μm). Bar graph on the *right* indicates the percent of positively stained area in brain regions of these mice (n = 3 technical replicates/genotype). Data are presented as mean ± SD from technical triplicates. *p* Values were determined by one-way ANOVA (hippocampus—F [2, 6] = 269.4, *p* < 0.0001; cerebellum—F [2, 6] = 269.4, *p* < 0.0001), followed by Tukey’s multiple comparisons test. ∗∗∗∗*p* < 0.0001. LCS, laforin cysteine-to-serine mutant; LKO, laforin KO; ns, nonsignificant.

To directly examine the impact of altered LCS activity in a biological context, we utilized an established LCS knock-in mouse model (45). Given the different biochemical properties of LCS and WT laforin, we first examined the age-dependent protein level of WT laforin and LCS compared with LKO as a control. Intriguingly, there was a decrease in laforin levels in the WT brain with age that was not observed with LCS. Instead, LCS levels remained significantly elevated (Fig. 3*A*). These data demonstrate that there is a unique stabilization of LCS in the brain *in vivo*, consistent with the observed biochemical increased stability and interactions of LCS *in vitro*. In contrast, a modest age-dependent decrease in both WT laforin and LCS was observed in the muscle (Fig. 3*B*). These results demonstrate unique properties of laforin in the brain, consistent with the central nervous system–centered disease pathology observed in LD.

The effect of these differences on brain glycogen accumulation was next measured. In contrast to LKO mice, LCS mice did not accumulate classical LBs (45), but evaluation of glycogen content in different brain regions revealed modest glycogen accumulation in the LCS mice compared with WT (Fig. 3*C*). In particular, increases in hippocampal glycogen were more pronounced.

### Perturbed brain metabolism reveals key aspects of laforin function

**Figure 4:**
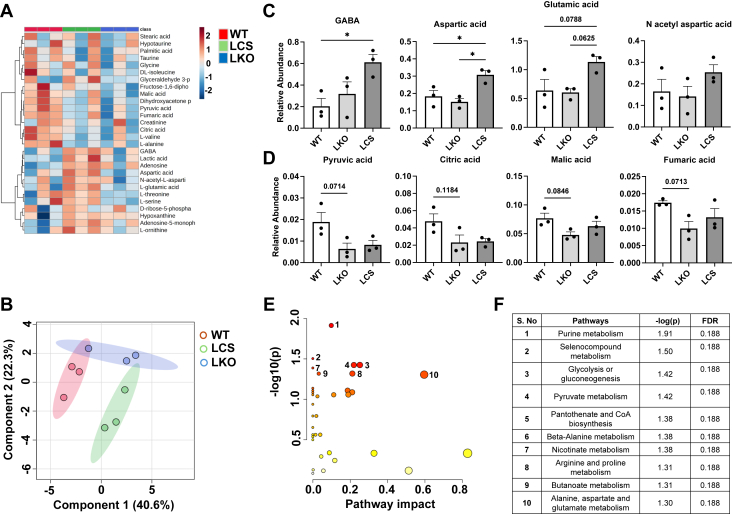
**Laforin phosphatase activity is needed to preserve brain metabolism.***A,* heatmap showing relative abundance of metabolites in the brain of 10-month-old WT, LKO, and LCS mice (n = 3 biological replicates/genotype) determined by GC–MS. *B,* multivariate analysis of polar metabolites identified in each genotype by partial least squares-discriminant analysis (PLS-DA) plot, demonstrating separation among WT, LKO, and LCS. *Shaded areas* represent the 95% confidence interval for each group. *C* and *D,* relative abundance of metabolites in the brain of 10-month-old WT, LKO, and LCS mice determined by GC–MS. *C,* GABA, glutamic acid, aspartic acid, and their *N*-acetyl-aspartic acid derivatives display distinct patterns in LCS mice compared with both WT and LKO. *D,* in contrast, selected glycolysis and TCA intermediates show no difference between LKO and LCS and trend similarly to WT. The data are average ± SD of three biological replicates and analyzed using one-way ANOVA (GABA—F [2, 6] = 5.784, *p* = 0.0398; aspartic acid—F [2, 6] = 9.587, *p* = 0.0135; glutamic acid—F [2, 6] = 5.23, *p* = 0.0484; NAA—F [2, 6] = 1.581, *p* = 0.2908; pyruvic acid—F [2, 6] = 4.51, *p* = 0.0638; citric acid—F [2, 6] = 3.60, *p* = 0.0937; malic acid—F [2, 6] = 3.509, *p* = 0.0979; and fumaric acid—F [2, 6] = 3.895, *p* = 0.0824, ∗*p* < 0.05). *E,* scatter plot representing the overall enrichment of metabolic pathways from metabolomic pathway analysis. The color of each circle (metabolic pathway) is related to significant changes (*p* value) obtained from enrichment analysis, and its size represents the fold enrichment score indicating metabolites in the corresponding pathway. *F,* list of significantly changing metabolic pathways (*p* < 0.05) with a false discovery rate value <0.2. GABA, gamma-aminobutyric acid; LCS, laforin cysteine-to-serine mutant; LKO, laforin KO; NAA, *N*-acetyl aspartic acid; TCA, tricarboxylic acid.

Previous studies have demonstrated that laforin loss is associated with metabolic defects (14, 72, 73). To determine the effect of LCS on metabolism *in vivo*, we performed targeted metabolomics analysis and profiled central metabolic pathways (glycolysis/tricarboxylic acid/amino acid metabolism) in the brain. We first assessed the global metabolomic effect of the LCS mutation in comparison to WT and LKO (Fig. 4, *A* and *B*). We observed that the metabolic profiles of LCS and LKO mice are distinct from WT, with significant changes in gamma-aminobutyric acid (GABA) and aspartic acid and increased trends in glutamic acid and *N*-acetyl aspartic acid (NAA) in LCS mice (Fig. 4, *A* and *C*). We also observed decreased trends in glycolysis and tricarboxylic acid cycle metabolites (*e.g*., pyruvic acid, citric acid, malic acid, and fumaric acid) in both LKO and LCS mice (Fig. 4, *A* and *D*). Global metabolic pathway analysis identified nucleotide (purine), glycolysis/pyruvic acid, CoA, and amino acids (arginine, proline, alanine, aspartic acid, and glutamic acid) as the prominently impacted metabolic pathways in LCS mice (Fig. 4, *E* and *F*). Together, these data demonstrate an altered metabolic status in the brain of both LCS and LKO mice.

The cellular energy and metabolic functions of both neurons and astrocytes are intimately coupled with the glutamic acid/GABA–glutamine cycle. This highly active and dynamic metabolic cycle involves astrocytes synthesizing glutamine and transporting it to neurons to replenish the pool of glutamic acid and GABA neurotransmitter. At the synapse, the secreted excitatory (glutamic acid) and inhibitory (GABA) neurotransmitters are taken up, metabolized, and recycled by the astrocytic/glial cells. Notably, the recycling of neurotransmitters was shown to be directly proportional to brain glucose oxidation (74). Interestingly, our data demonstrate that compared with WT, GABA and glutamic acid levels are unchanged in the LKO brain but increased in the LCS brain (Fig. 4*C*). Neuronal glutamic acid serves as a key substrate in transamination reactions to produce aspartic acid, a critical precursor for NAA synthesis. Strikingly, aspartic acid is significantly increased in the LCS brain (Fig. 4*C*), suggesting a distinct utilization pattern of neuronal glutamic acid for the synthesis of aspartic acid, and potentially NAA, in the LCS brain compared with the WT and LKO brain. These data establish that laforin phosphatase activity is a key determinant of glycogen homeostasis and broader metabolic equilibrium in the brain.

## Discussion

In the present study, we define the unique biochemical properties of the glycogen phosphatase laforin when the catalytic cysteine is mutated to serine (LCS). *In vitro,* LCS displays loss of catalytic activity and a number of unique changes compared with the WT protein: LCS binds with micromolar affinity to phosphate; displays a much greater stabilization in the presence of glycogen and long-chain oligosaccharides; and it binds with higher affinity and positive cooperativity to DP24, although these effects are attenuated in high phosphate. *In vivo,* we found that a mouse model expressing LCS exhibits increased glycogen levels in specific brain regions and aberrant brain metabolism.

Like laforin, other PTP C/S mutants are dynamically different from the WT proteins (58, 75), and C/S and D/A mutants are often used for substrate trapping (34). The enhanced binding of LCS to long glucans with phosphate in the active site may be analogous to the observed binding of PTP mutants to their phosphorylated protein substrates, in this case, a glycogen trap (76, 77). Our results suggest reduced conformational dynamics in LCS and the favoring of a “closed” conformation of the D-loop, which is also consistent with the bound phosphate at the active site. We previously observed similar changes in solvent accessibility of the D-loop, recognition domain, PTP-loop, and R-motif *via* HDX when WT laforin was bound to glycogen (30). Therefore, WT binding to glycogen may produce similar conformational shifts as LCS bound to phosphate. Since LCS also displayed increased interaction with PTG and malin in yeast two-hybrid assays, these conformational changes seem to also be associated with increased protein–protein interactions.

We previously reported that most LD-causing mutations affect stability, carbohydrate binding, and/or protein–protein interactions, with variable effects on glycogen phosphatase activity (33). These data suggest that laforin-binding activities may be more critical than catalysis for preventing LB formation and LD. The enhanced interaction of LCS with malin and PTG suggests that carbohydrate binding and protein–protein interactions may be coupled, supporting the model that laforin acts as a scaffolding protein (45, 61, 78, 79, 80). LCS may be more effective than WT laforin at recruiting or retaining malin and PTG on the glycogen molecule at locations where there are long, phosphorylated glucan chains present. This recruitment could promote malin-directed ubiquitination of glycogen-associated substrates, leading to glycogen remodeling. These events could also impact metabolic regulation, which is an area of active investigation.

Interestingly, hyperphosphorylated glycogen is also present in malin-deficient mouse models of LD, despite the presence of fully functional laforin (44, 81). Recent proteomic analyses from coimmunoprecipitation of myc-tagged malin identified a protein complex between malin and laforin with several additional interacting proteins involved in glycogen metabolism: glycogen debranching enzyme (Agl), glycogen synthase (Gys1), glycogen phosphorylase (Pygm), glycogenin (Gyg1), starch-binding domain–containing protein 1 (Stdb1), and the protein phosphatase 1 regulatory subunit Pp1r3a (45). These interactions appear to be direct. Additional studies are required to understand the nature of macromolecular complexes centered on laforin in different subcellular contexts, including differential recruitment and competition among binding partners.

Glycogen hyperphosphorylation in the absence of laforin has been implicated as a driver of the biogenesis of poorly branched and insoluble LBs. Both LD mice and patients also exhibit perturbed cerebral metabolism (14, 73, 82) that has potential implications in altered synaptic functions. However, the direct cause of the cerebral metabolic disruption in LD is not well understood. The unique properties of LCS suggest that laforin phosphatase activity may be coupled to protein–protein interactions in the context of normal glycogen metabolism. Even in the absence of abundant LBs, as observed in the LCS mouse model, the lack of laforin phosphatase activity may lead to energy stress, characteristic of laforin-deficient LD. Therefore, both the phosphatase activity of laforin and its role as an adaptor protein play a role in normal glycogen metabolism, and the multiple activities of laforin should be carefully considered when examining the pathological mechanisms of LD.

Laforin dysfunction in LCS mice is related to both loss and gain of function(s). Glycogen hyperphosphorylation and/or protein–protein interaction/s could drive abnormal cerebral metabolism. It remains to be determined whether these possibilities are interconnected or independently control cerebral metabolism. Also, given the differences observed between LCS protein levels in brain and muscle, it may be that different brain regions (*i.e*., hippocampus, cerebellum, cortex, etc.) and/or individual cell types in the brain (*e.g.*, neurons *versus* astrocytes) may exhibit different perturbations. Indeed, it was previously reported that the transgenic expression of LCS on the LKO background led to an improvement in memory measured by an object recognition test, suggesting that some neuronal function is corrected with LCS (43). However, LBs have different consequences in different cell types (15), and the LCS mice may have some cerebral dysfunction as a result of glycogen hyperphosphorylation and altered metabolic pathways.

LD patients experience progressive myoclonus and generalized tonic–clonic seizures, and older LD mouse models recapitulate these symptoms to varying degrees, including spontaneous tonic–clonic seizures and heightened sensitivity to epileptogenic agents, such as pentylenetetrazole and kainate (83, 84, 85). In both LD patients and mice, the epileptic activity likely reflects an imbalance between excitatory (glutamic acid and aspartic acid) and inhibitory (GABA) amino acid neurotransmitters. Indeed, LD astrocytes exhibit compromised glutamic acid uptake, leading to higher levels of this neurotransmitter in the brain parenchyma (86, 87). The metabolic link between glutamic acid and GABA is maintained through the glutamic acid/GABA–glutamine cycle. NAA, synthesized from aspartic acid and acetyl-CoA in neuronal mitochondria, serves as a marker of neuronal health and as a reservoir for replenishing glutamic acid (88). A recent study in LD patients with myoclonus or convulsive seizures revealed significantly higher levels of excitatory amino acids (glutamic acid and glutamine) and lower levels of the inhibitory neurotransmitter GABA, and also NAA, potentially linking these metabolic alterations with neuronal hyperexcitability and neurodegeneration in LD (82). Our findings further strengthen the link between laforin phosphatase activity and broader brain metabolism. The altered metabolic profile of LCS mice reveals that glycogen phosphorylation influences central carbon metabolism and neurotransmitter balance. These results provide important insights regarding the molecular basis of LD and the integral role of glycogen in brain metabolism.

## Experimental procedures

### Cloning, protein expression, and purification

WT and mutant human laforin recombinant proteins were produced as His6-tagged fusions from pET28b (Novagen) in BL21-Codon Plus *Escherichia coli* cells (New England BioLabs) and purified by immobilized metal affinity chromatography and size-exclusion chromatography as previously described (30, 33). LCS was generated by site-directed mutagenesis (QuikChange Lightning, Agilent; Q5 Site-Directed Mutagenesis, New England BioLabs; GENEWIZ Site-Directed Mutagenesis). Purity of proteins was confirmed by SDS-PAGE with Coomassie staining.

### Phosphate detection and dephosphorylation assays

Glycogen purified from rabbit muscle has higher levels of phosphate than commercial glycogen and was therefore used for dephosphorylation assays (23). Dephosphorylation assays were performed in phosphatase buffer (100 mM sodium acetate, 50 mM Bis–Tris, 50 mM Tris–HCl, pH 6.5) containing 2 mM DTT, 2.5 μg enzyme, and 10 mg/ml rabbit muscle glycogen as substrate, at 25 °C for 30 min as previously described (33, 41). Phosphate release during reaction was quantified using the P_i_ ColorLock Gold Phosphate Detection system (Innova Biosciences), a commercial malachite green assay–based reagent for detecting inorganic phosphate.

### Differential scanning fluorimetry

The DSF experiments were performed as described previously (30, 33). The substrate DP24 refers to a mixture of linear oligosaccharides (10–40 glucosyl units) with an average length of 24 units (Elicityl; catalog no.: GLU310) (30). Human laforin C266S, buffers, water, rabbit liver glycogen (Sigma; catalog no.: G8876), and DP24 were always preincubated with PiBind resin (Novus Biologicals; catalog no.: 501-0015) to remove any contaminating phosphate before the assay was performed. Each reaction mix contained 2 μM of purified protein, DP24, or rabbit liver glycogen as substrates and 5× SYPRO Orange Protein Gel Stain. For phosphate-binding assays, phosphate was added in the reaction mix from a 0.05 M K_2_HPO_4_/KH_2_PO_4_ stock. Melting was monitored over a temperature range of 20 to 90 °C, with a ramp of 1 °C per 50 s. The *T*_m_ was determined by applying a Gaussian fit to the first derivative of the melting curve. Data analysis and binding curve fitting were performed using GraphPad Prism 6 software (GraphPad Software, Inc).

### Hydrogen–deuterium exchange

For HDX analysis, 99.7% coverage was obtained with 310 high-quality matched peptides (Fig. S3). Quenching conditions for optimal sequence coverage of laforin were previously established as 0.08 M GuHCl, 0.1 M glycine, 16.6% glycerol, pH 2.4 (30). Functional HDX experiments were initiated by dilution of 3 μl of stock solution (WT or LCS at 1 mg/ml) into 9 μl of D_2_O buffer (8.3 mM Tris, 150 mM NaCl, and pD_READ_ 7.2), and the mixture was incubated at 0 °C. The exchange reactions were quenched at various times (10, 100, 1000, 10,000, and 100,000 s) by the addition of 18 μl of the optimal quench solution, and the quenched samples were flash frozen with dry ice. Undeuterated and equilibrium-deuterated control samples were also prepared as previously described (30, 36). All frozen samples were passed over an immobilized pepsin column (16 μl) at a flow rate of 25 μl/min, and digested peptides were collected on a C18 trap column (Optimize Tech, Opti-Trap, 0.2 × 2 mm) for desalting. Peptide separation was performed on a C18 reverse-phase column (Agilent, Poroshell 120, 0.3 × 35 mm, 2.7 μl) with a linear gradient of 8% to 48% B over 30 min (A: 0.05% TFA in H_2_O; B: 80% acetonitrile, 0.01% TFA, and 20% H_2_O). MS analysis was performed on the Orbitrap Elite mass spectrometer (Thermo Fisher Scientific), adjusted for HDX experiments (89). The resolution of the instrument was set at 120,000 at *m/z* 400. Proteome Discoverer software (version 1.3; Thermo Scientific) was used to identify the sequence of the digested peptide ions from their MS/MS data. HDXaminer (Sierra Analytics) was utilized to confirm the peptide identification and calculate the centroids of isotopic envelopes of all the peptides. The level of deuterium incorporation of each peptide was calculated by applying back-exchange correction (90).

### Yeast two-hybrid assays

For yeast two-hybrid assays, laforin WT and LCS were expressed in the pEG202 vector encoding a LexA-fusion protein, whereas malin and PTG were expressed in the pACT2 vector encoding Gal4 activation domain fusion proteins. The experiments were performed as previously described (33). Briefly, *Saccharomyces cerevisiae* transformed with the indicated plasmids were grown in selective synthetic complete medium, and the transformants were screened for β-galactosidase activity using a filter lift assay. To quantify protein interactions, four to six independent transformants were permeabilized, and the β-galactosidase activity was measured in yeast cell extract and expressed in Miller Units.

### Mouse lines and tissue harvesting

The laforin LKO was previously described (91). The laforin C265S knock-in (LCS) mouse model used was generated by mutating cysteine 265 of the mouse laforin gene to serine as previously described (45). All animal studies were conducted in accordance with federal guidelines and were approved by the Institutional Animal Care and Use Committees of Indiana University, School of Medicine. The mice were housed in a 12:12 h light–dark cycle and were given ad libitum access to food and water. The mice were euthanized by cervical dislocation, decapitated, and the heads were plunged into liquid nitrogen and stored at −80° C until further use. For brain tissue collection, the skulls were dissected in liquid nitrogen, the brains were removed, and the brains were pulverized to 5 μm particles in liquid nitrogen using a Freezer/Mill Cryogenic Grinder (SPEX SamplePrep), and the powdered tissue was stored at −80 °C.

### Western blot analysis

Mouse tissue samples were prepared and analyzed according to a previously described method (45). Briefly, 50 to 100 mg of powdered frozen skeletal muscle or brain from 4.5- or 12-month-old mice were homogenized for 25 to 30 s with a Tissue Tearer in 15 volumes of homogenization buffer containing 50 mM Tris–HCl, pH 7.8, 10 mM EDTA, 2 mM EGTA, 100 mM NaF, 10 μg/ml leupeptin, 1 mM benzamidine, 0.1 mM *N*α-p-tosyl-l-lysine chloromethyl ketone, 0.5 mM PMSF, 1 mM sodium orthovanadate, 0.4% (v/v) β-mercaptoethanol, and 0.2% (v/v) Triton X-100. The homogenates were centrifuged at 6000*g* for 10 min. For immunoblotting, 30 to 40 μg of soluble protein were analyzed. Samples were separated on 10% SDS-PAGE gels at 200 V and transferred onto a nitrocellulose membrane at 100 V for 1.5 h or at 20 V for 20 h at 4 °C. The membranes were stained with Ponceau Red, destained, and blocked using Tris-buffered saline with 0.1% Tween-20 detergent and 5% nonfat dry milk for 2 h at room temperature. The membranes were probed with antibodies against laforin or GAPDH at 4 °C for 20 h, followed by incubation with anti-mouse/rabbit horseradish peroxidase–conjugated secondary antibody (Sigma) for 1 h at room temperature. The anti-laforin antibody was from Abnova (catalog no.: H00007957-M02), and its specificity was established in a previous study (45). The anti–glyceraldehyde-3-phosphate dehydrogenase antibody was from Biodesign International (catalog no.: H86504M). The binding of the antibody was detected by enhanced chemiluminescence (Pierce ECL Western Blotting Substrate; Thermo Scientific).

### Immunohistochemical analysis

For immunohistochemical analyses, WT, LKO, and LCS mice were euthanized at 12 months of age by cervical dislocation, and the brain was rapidly dissected and fixed in formalin. Formalin-fixed brains were embedded in paraffin blocks. Samples were sectioned, placed on slides, and stained with IV58B6 anti-glycogen antibody, whose specificity was established earlier (92, 93). Slides were scanned using a Zeiss Axio Scan Z1 and then loaded into HALO v3.3.2541.345 software designed by Indica Labs. HALO software was used to annotate brain regions, including the hippocampus and the cerebellum, and then Indica Labs Area Quantification, version 1.0, was used to quantify the percent positive stain.

### GC–MS metabolomics analysis

GC–MS metabolomics experiments were performed as previously described (14, 15). Briefly, 10-month-old WT, LCS, and LKO mice were sacrificed by spinal dislocation, and the brain was removed, washed in PBS and diH_2_O, and then snap frozen in liquid nitrogen. The frozen tissue was then pulverized using a Freezer/Mill Cryogenic Grinder. The pulverized tissue (20 mg) from each brain was added to 50% methanol for metabolite extraction. The samples were separated into the polar and nonpolar fractions through centrifugation. Both fractions were removed and dried by vacuum centrifuge at 10^−^^3^ mBar. Dried samples were then derivatized by dissolving the sample in 20 mg/ml methoxyamine in pyridine and incubating at 60 °C for 90 min. Samples were then treated with *N*-methyl-*N*-(trimethylsilyl)trifluoroacetamide, thoroughly mixed, and incubated at 37 °C for 30 min. The mixture was then transferred to a V-shaped glass chromatography vial and analyzed by GC–MS. The data were normalized to amino acids in the nonpolar fraction and processed using Agilent Mass Hunter, and statistics were run using MetaboAnalyst (https://www.metaboanalyst.ca/).

### Statistics

Data from metabolomics analysis are presented as mean ± SD and were processed using GraphPad Prism. Statistical significance was evaluated using one-way or two-way ANOVA and considered significant at *p* < 0.05.

## Data availability

All data are included in the article or the supporting information. Raw data are available upon request.

## Supporting information

This article contains supporting information (30, 94, 95).

## Declaration of generative artificial intelligence and artificial intelligence–assisted technologies in the writing process

During the preparation of this work, the authors used ChatGPT in order to enhance language clarity. After using this tool, the authors reviewed and edited the content as needed and take full responsibility for the content of the publication.

## Conflict of interest

P. J. R., A. D. R., M. S. G., and R. C. S. have received research support and/or consultancy fees from Maze Therapeutics. R. C. S. is a member of the Medical Advisory Board for Little Warrior Foundation, and M. S. G. is a member of the Science Advisory Board for Chelsea’s Hope Lafora Research Foundation, Glut1-Deficiency Syndrome Foundation, and the Adult Polyglucosan Body Disease Foundation. All other authors declare that they have no conflicts of interest with the contents of this article.
