# Supplementary material for: The biochemical dynamics of the glycogen phosphatase laforin directly impact brain metabolism
Source: J Biol Chem. 2025 Dec 22;302(2):111097. doi: 10.1016/j.jbc.2025.111097 (PMC12834914; doi:10.1016/j.jbc.2025.111097)
Supplement: Supporting information [file mmc1.docx]

**SUPPORTING INFORMATION**

**TITLE: The biochemical dynamics of the glycogen phosphatase laforin directly impacts brain metabolism**

M. Kathryn Brewer^1,7^, Katherine J. Donohue^1,7^, Pankaj K. Singh^2,7^, Madushi Raththagala^6^, Zoe R. Simmons^1^, Jeremiah L. Wayne^1^, Sheng Li^3^, Rosa Viana^4^, Dyann M. Segvich^5^, Christopher J. Contreras^5^, Alex R. Cantrell^2^, Pascual Sanz^4^, Ramon C. Sun^2^, Craig W. Vander Kooi^2^, Peter J. Roach^5†^, Anna DePaoli-Roach^5^, and Matthew S. Gentry^2*^

**Supporting methods**

*Phosphate determination in purified LCS*

To measure phosphate in purified laforin proteins, we added malachite green reagent to 100 μg of native and boiled WT, LCS (C266S), and C266A laforin. After malachite green was added, the samples were centrifuged, and the soluble fraction was added to a 96-well plate for spectrophotometric quantification (**Fig. S1A**). While WT and C266A contained no phosphate, phosphate was readily detected in both native and boiled C266S samples (**Fig. S1B, C**). Malachite green reagent was also added to the remaining precipitated protein pellets. The pellets from the C266S samples that were not boiled stained more intensely, while the boiled C266S samples stained weakly. These data suggest the phosphate is tightly bound in a pocket of the protein, and the rapid denaturation caused by the acidic reagent causes the bound phosphate to precipitate with the collapsed protein. WT and C266A showed no phosphate, while C266S had approximately 0.344 nmol phosphate per nmol protein (**Fig. S1C**). We also confirmed that all catalytic mutants exhibited no phosphatase activity (**Fig. S1D**).

*Phosphate stripping of LCS*

To identify the source of the scavenged phosphate, we performed a phosphate-strip experiment based on a published method used to remove phosphate from the well-known bacterial phosphate-scavenger PstS (2). To remove possible sources of phosphate contamination, all buffers were pre-incubated with PiBind (Novus Biologicals #501-0015), a commercial resin designed to remove contaminating phosphate from buffers and prevent high background in phosphate-sensitive enzymatic reactions. After expression of LCS was induced, *E. coli* cells were harvested by centrifugation, resuspended in phosphate-free buffer, and lysed. Insoluble protein in the lysate was pelleted by centrifugation. The clarified lysate was then divided into two aliquots (“control” and “strip”), which were separately loaded onto a nickel column for affinity chromatography, and the flow-through was collected for each. After loading, the control sample was washed with 12 column volumes and then eluted with 300 mM imidazole, according to our standard protocol. The strip sample was washed with 70 column volumes and then eluted. Samples taken from each step of the purification show that the lysate and column flow-through fractions are phosphate-rich (saturating the malachite green assay), and that the eluted proteins from both strip and control samples contained equal amounts of phosphate (**Fig. S1E**). When normalized to protein content, phosphate detected was approximately 0.5 nmol phosphate per nmol protein.

**Fig. S1. LCS (C266S) scavenges phosphate from *E. coli.*** (A) Schematic of the LCS (C266S) phosphate detection experiment. (B) Visualization of phosphate in the soluble and insoluble protein fractions after the addition of malachite green reagent. (C) Quantification of phosphate in WT and catalytic mutants. (D) Glycogen phosphatase activity of catalytic mutants. (D) Visualization and quantification of phosphate during “control” and “strip” purifications of LCS from *E. coli.* Flow through (FT) and elution are from the immobilized nickel affinity chromatography step. Three wells represent the technical replicates used for the assay. For (C), (D) and (E), average of technical triplicates ± SD are shown.

**Fig. S2. WT and LCS binding to phosphate and glucans.** (A) T_m_ binding curves of WT versus LCS with phosphate, corresponding to ΔT_m_ data in Fig. 1B. (B) T_m_ binding curves of WT versus LCS with glycogen or DP24 and 0, 0.1 or 1 mM phosphate added. (B) ΔT_m_ binding curves of WT versus LCS with glycogen or DP24 and 0, 0.1 or 1 mM phosphate added. In (B) and (C), T_m_ and ΔT_m_ curves for 0 mM phosphate correspond to the same data shown Fig. 1C and D (for glycogen and DP24, respectively). Data represents average ± SD.

**Fig. S3. Hydrogen deuterium exchange (HDX) mass spectrometry peptide map.** 99.7% sequence coverage of laforin by pepsin digested peptides. Residues 2-328 were covered by at least one peptide. A total of 310 high-quality peptides were identified.

|  | **Phosphate** | | **Glycogen**  **(no phosphate)** | | **Glycogen + 0.1 mM phosphate** | | **Glycogen + 1 mM phosphate** | | **DP24**  **(no phosphate)** | | **DP24 + 0.1 mM phosphate** | | **DP24 + 1 mM phosphate** | |
| --- | --- | --- | --- | --- | --- | --- | --- | --- | --- | --- | --- | --- | --- | --- |
| WT vs LCS | WT | LCS | WT | LCS | WT | LCS | WT | LCS | WT | LCS | WT | LCS | WT | LCS |
| B_max,app_ (ºC) | ND | 9.459 ± 0.3296 | 5.407 ± 0.2208 | 16.51 ± 0.7847 | 6.055 ± 0.2678 | 10.42 ± 2.214 | 7.010 ± 0.3794 | 2.861 ± 0.1885 | 9.182 ± 0.3039 | 17.80 ± 0.2262 | 9.730 ± 0.3458 | 13.37 ± 0.2735 | 9.872 ± 0.3725 | 18.84 ± 0.5054 |
| B_max,app_ (ºC) (corrected) | N/A | N/A | N/A | N/A | N/A | 15.18 | N/A | 9.716 | N/A | N/A | N/A | 18.13 | N/A | 25.73 |
| Hill coefficient | ND | 0.4905 ± 0.03678 | 0.9633 ± 0.1076 | 0.7495 ± 0.03306 | 0.7914 ± 0.07306 | 0.7760 ± 0.1094 | 0.6111 ± 0.04877 | 1.530 ± 0.2838 | 0.4946 ± 0.02105 | 1.335 ± 0.08532 | 0.4754 ± 0.01986 | 0.9930 ± 0.05507 | 0.4787 ± 0.02149 | 0.5556 ± 0.008797 |
| K_d,app_ (mM) | ND | 0.05471 ± 0.01225 | 0.6001 ± 0.09205 | 2.951 ± 0.4405 | 0.7724 ± 0.1347 | 6.004 ± 3.514 | 1.050 ± 0.2506 | 1.247 ± 0.1984 | 0.4092 ± 0.07040 | 0.04648 ± 0.002606 | 0.5048 ± 0.09431 | 0.3474 ± 0.02582 | 0.4927 ± 0.09746 | 5.486 ± 0.5468 |
| DF | 9 | 9 | 39 | 36 | 39 | 36 | 39 | 36 | 45 | 45 | 45 | 45 | 45 | 45 |
| R^2^ | 0.5842 | 0.9951 | 0.9618 | 0.9955 | 0.9727 | 0.9646 | 0.9788 | 0.9428 | 0.9933 | 0.9886 | 0.9937 | 0.9908 | 0.9927 | 0.9993 |

**Table S1. Apparent binding parameters from DSF experiments.** All data were fit using the one site specific binding model (with Hill slope). Best-fit values are shown ± standard error. Note that binding fits are calculated based on ΔT_m_ data. For glycogen or DP24 titrations with 0.1 mM or 1 mM phosphate, the calculated B_max,app_ does not take into account the baseline temperature shift (ΔT_m_) for LCS that occurs in presence of phosphate in the absence of glucan (ΔT_m_ = 4.755 ºC with 0.1 mM phosphate and ΔT_m_ = 6.855 ºC with 1 mM phosphate). Therefore, “corrected” B_max,app_ values are also shown that represent the total maximum ΔTm (B_max,app_ + baseline ΔT_m_). ND = not determined due to poor fit (no binding); N/A = not applicable.

**REFERENCES**

1. Raththagala, M., Brewer, M. K., Parker, M. W., Sherwood, A. R., Wong, B. K., Hsu, S., Bridges, T. M., Paasch, B. C., Hellman, L. M., Husodo, S., Meekins, D. A., Taylor, A. O., Turner, B. D., Auger, K. D., Dukhande, V. V., Chakravarthy, S., Sanz, P., Woods, V. L., Jr., Li, S., Vander Kooi, C. W., and Gentry, M. S. (2015) Structural Mechanism of Laforin Function in Glycogen Dephosphorylation and Lafora Disease. *Mol Cell* **57**, 261-272

2. Neznansky, A., Blus-Kadosh, I., Yerushalmi, G., Banin, E., and Opatowsky, Y. J. T. F. J. (2014) The Pseudomonas Aeruginosa Phosphate Transport Protein Psts Plays a Phosphate-Independent Role in Biofilm Formation. **28**, 5223-5233

3. Hutchison, A. J. (2009) Oral Phosphate Binders. *Kidney international* **75**, 906-914
